# The SIX1/LDHA Axis Promotes Lactate Accumulation and Leads to NK Cell Dysfunction in Pancreatic Cancer

**DOI:** 10.1155/2023/6891636

**Published:** 2023-03-08

**Authors:** Wanli Ge, Lingdong Meng, Shouji Cao, Chaoqun Hou, Xiaole Zhu, Dongya Huang, Qiang Li, Yunpeng Peng, Kuirong Jiang

**Affiliations:** ^1^Pancreas Center, The First Affiliated Hospital of Nanjing Medical University, Nanjing, China; ^2^Pancreas Institute, Nanjing Medical University, Nanjing, China; ^3^Nanjing Medical University, Nanjing, China; ^4^Thyroid Surgery, The First People's Hospital of Lianyungang, Lianyungang, China

## Abstract

**Background:**

Pancreatic cancer (PC) is a malignant cancer with poor prognosis and high mortality rate. Sine oculis homeobox homolog 1 (SIX1) participates in the development of many cancers. However, the function of SIX1 in PC is not fully understood.

**Methods:**

SIX1 expression was determined using immunohistochemistry in PC tissues and cell lines. Glucose consumption, lactate production, and ATP assays were used to detect the function of SIX1. PC cells and NK cells were cocultured to study the effect of SIX1 overexpression in PC cells on NK cell function. Chromatin immunoprecipitation (ChIP) assays were used to study the relationship between SIX1 and lactate dehydrogenase A (LDHA). A series of in vitro and in vivo assays were further applied to elucidate the important role of the SIX1/LDHA axis in metabolism and NK cell dysfunction in PC.

**Results:**

SIX1 was significantly upregulated in PC tissue; SIX1 overexpression promoted the glycolysis capacity of PANC-1 and CFPAC-1 cells and resulted in NK cell dysfunction after the NK cells had been cultured with PC cells. LDHA inhibitor partially restored the promotion of PC caused by SIX1 overexpression. According to ChIP assays, SIX1 directly binds to the LDHA promoter region. Moreover, LDHA inhibitor and lactate transporter blocker treatment promoted the function of NK cells cocultured with PC cells. In vivo experiments yielded the same results.

**Conclusion:**

The SIX1/LDHA axis promotes lactate accumulation and leads to NK cell dysfunction in PC.

## 1. Introduction

Pancreatic cancer (PC) is a malignant tumor with a high mortality rate. The incidence of PC in the United States is gradually increasing, and the five-year survival rate is only 9% [1, 2]. PC metabolism is characterized by a high rate of glycolysis, which produces energy to maintain the proliferation, migration, and invasion of PC cells [3–7]. Moreover, studies have found that there are a variety of mechanisms mediating the effect of glycolysis on oncogenesis and tumor development [8–10]. Therefore, it is crucial that we have a deep understanding of the effects of glycolysis on PC.

An increasing number of studies have found that SIX1 plays an important role in tumor glycolysis, thereby promoting tumor progression [11, 12]. Glycolysis in cancer tissues often leads to lactate accumulation. The increased production of lactate is related to the malignant progression of cancer and poor patient survival outcomes [13–15]. The relationship between the immune system and LDH is complex. LDH is released from tumors and it is one of the markers for progression in many tumors [16]. This occurs not only because glycolysis provides more energy to cancer cells but also because the lactate produced by glycolysis can directly affect the function of immune cells [17, 18]. Studies have found that lactate can be a signaling molecule that plays an important role in the regulation of the tumor immune response and cell-to-cell communication [19, 20]. Apart from LDH, the tumor also secretes other inflammatory mediators that have a negative effect on the cells of the immune system [21, 22]. As an important part of tumor immunity, NK cells have no MHC-mediated restriction of their killing activity and participate in the body's first line of defense against malignant cells [23, 24]. NK cell dysfunction in PC is an important cause of its malignant progression [25]. Recent studies have shown that lactate can indirectly inhibit NK cell function by regulating other immunosuppressive cells (for example, by stimulating the recruitment of myeloid-derived suppressor cells (MDSCs) or by increasing the metabolic advantage of Tregs) [26, 27]. However, no research has proven whether glycolysis of tumor cells can inhibit NK cell function in PC.

New methods of treating tumors that target lactate production or lactate transport are continually emerging. Our research reveals the regulatory effect of the SIX1/LDHA axis on lactate and explores the negative effect of the SIX1/LDHA axis on the function of NK cells. Therefore, targeting the SIX1/LDHA axis to regulate the effect of lactate on NK cell function may become a new method of immunotherapy for PC.

## 2. Materials and Methods

### 2.1. Cell Culture

Four PC cell lines (PANC-1, BXPC-3, CFPAC-1, and MIAPACA-2) and the normal human pancreatic ductal cell line HPNE were purchased from the Cell Bank of Type Culture Collection of the Chinese Academy of Sciences in Shanghai, China. The cells were cultured in Dulbecco's modified Eagle's medium (Life Technologies, USA) containing 10% fetal bovine serum (Wisent, Canada) at 37°C in a humidified incubator with 95% air and 5% CO_2_. To investigate the effects of the SIX1/LDHA axis on NK cell function, 3 × 10^5^ NK cells were cocultured with 5 × 10^5^ PANC-1 or CFPAC-1 cells per well in 6-well plates in the presence or absence of 10 *μ*M GNE-140 (a specific blocker for LDHA) or 10 *μ*M 7ACC1 (a specific blocker for MCT1 and MCT4).

### 2.2. Patients and Pancreatic Tissues

One hundred pairs of pancreatic tumor tissue and adjacent nontumor tissue samples were obtained from patients who underwent surgery at the First Affiliated Hospital of Nanjing Medical University, China. No antitumor therapy treatment was given to the patients before surgery. Histopathological diagnoses were confirmed by two experienced pathologists. Written informed consent was obtained from the patients or their relatives, and the study was approved by the Ethics Committee of the First Affiliated Hospital of Nanjing Medical University (2021-SRFA-236).

### 2.3. Peripheral Blood Mononuclear Cell (PBMC) Isolation and NK Cell Expansion

Peripheral blood was collected from healthy males with written informed consent and diluted with PBS. PBMCs were separated using the Lymphoprep density gradient centrifugation method. The isolated PBMCs were used to expand NK cells using the Natural Killer Cells Culture Kit Plus (Dakewe, China) according to the instructions. The final purity of the NK cells (CD3-, CD16+, and/or CD56+) was >90% as quantified by flow cytometry.

### 2.4. Western Blotting

The proteins were subjected to 8–12% SDS-PAGE and transferred to a PVDF membrane (Bio-Rad, USA). The membranes were then incubated with 5% nonfat milk in Tris-buffered saline containing 0.1% Tween (TBST) at room temperature for 2 h and incubated with primary antibodies at 4°C overnight. Following washing with TBST (15 min) three times, the membranes were incubated with HRP-conjugated anti-mouse or anti-rabbit IgG secondary antibodies at room temperature for 2 h. The protein expression levels were visualized with Immobilon Western Chemilum HRP substrate (Merck Millipore, Darmstadt, Germany) using an enhanced chemiluminescence detection system. The primary antibodies against granzyme B, perforin, and MCT4 were obtained from Proteintech (Wuhan, China); the primary antibodies against LDHA, SIX1, and TNF-*α* were obtained from Servicebio (Wuhan, China); and the primary antibody against MCT1 was obtained from Beyotime Biotechnology (Shanghai, China).

### 2.5. Immunohistochemistry (IHC)

SIX1 and LDHA expressions in the PC tissue microarray were determined by IHC. IHC was performed according to the manufacturer's protocol. In brief, the samples were rehydrated with ethanol after dewaxing with xylene. The tissue microarrays were incubated with 3% H_2_O_2_ for 5 min and sodium citrate buffer (pH 6.0) for 20 min. Finally, the sections were incubated with polyclonal antibodies against SIX1 and LDHA overnight at 40°C, followed by incubation with the secondary antibodies. The IHC score was calculated to assess the expression level based on the staining intensity and the positive cell ratio as follows: IHC score = (percentage of cells with weak staining × 1) + (percentage of cells with medium staining × 2) + (percentage of cells with strong staining × 3).

### 2.6. Transient Transfection of siRNAs and Plasmids

SIX1 overexpression and empty vector plasmids were synthesized by GenePharma (Shanghai, China). SIX1-targeting small interfering RNA (siRNA) (sense 5′-GUCAGCAACUGGUUUAAGATT-3′ and antisense 5′-UCUUAAACCAGUUGCUGACTT-3′) and control siRNA (sense 5′-UUCUCCGAACGUGUCACGUTT-3′ and antisense 5′-ACGUGACACGUUCGG AGAATT-3′) were purchased from GenePharma (Shanghai, China). Lipofectamine 3000 (Life Technologies) was used to perform transient transfections according to the manufacturer's instructions. qRT-PCR and western blotting were carried out to detect the expression of the target gene.

### 2.7. RNA Isolation and Quantitative RT-PCR

Total RNA was extracted from PANC-1 and CFPAC-1 cells using TRIzol reagent (Life Technologies, USA) according to the instructions. qRT-PCR was performed using FastStart Universal SYBR Green Master Mix (Roche, Switzerland). The 2^−*ΔΔ*CT^ method was used to calculate the relative expression of target genes. The primers are shown in Supplementary Table S1.

### 2.8. ChIP

ChIP assays were performed using the EZ ChIP kit (Millipore, Germany) according to the manufacturer's instructions. Lysates of PANC-1 or CFPAC-1 cells were incubated with antibodies against SIX1. The probable binding motifs for SIX1 (<2000 bp proximal promoter region) were assessed using the JASPAR database (http://jaspar.genereg.net/), and the two most likely binding motifs lie within the -1902 to -1913 and -1759 to -1770 LDHA promoter regions. The length of the ChIP qPCR products containing promoter sites 1 and 2 was 188 bp and 265 bp, respectively. The primers are shown in Supplementary Table S1.

### 2.9. Flow Cytometric Analysis

After cocultivating NK cells and PC cells with different treatments for three days, NK cells were collected and washed twice with PBS, and then, anti-human CD3-PerCP/Cyanine5.5, CD16-PE, CD56-PE, NKG2D-APC, NKp46-PE/Cyanine7, and DNAM-1-FITC antibodies were added (BioLegend, San Diego, CA, USA) according to the instructions. The cells were incubated for 20 min in the dark and washed twice with PBS. For the detection of NK cells in mouse peripheral blood, 100 *μ*l mouse peripheral blood was collected, and anti-mouse CD3-PerCP/Cyanine5.5, NK1.1-PE-Cyanine7, NKG2D-APC, NKp46-FITC, and DNAM-1-PE antibodies were added (BioLegend, San Diego, CA, USA) and incubated at room temperature for 20 minutes in the dark. Then, 2 ml RBC lysis solution was added to each tube, mixed well, and incubated for 15 min. Then, the samples were centrifuged, the supernatant was discarded, and the cells were washed twice with PBS. All samples were then analyzed by multicolor flow cytometry (BD, USA).

### 2.10. Glucose Consumption, Lactate Production, and ATP Assays

PANC-1 and CFPAC-1 cells were seeded into 6-well plates and cultured under different treatment conditions. The Glucose Assay Kit (Abcam, USA) and Lactate Assay Kit (Abcam, USA) were used to test glucose consumption and lactate production following the manufacturer's protocol, respectively. An ATP assay kit (Beyotime, China) was used to measure ATP levels according to the manufacturer's instructions. The relative levels of glucose consumption, lactate production, and ATP in all treated groups were normalized to the control group.

### 2.11. Animal Study

Female C57BL/6 mice (8–12 weeks old) were purchased from the Animal Center of Nanjing Medical University. PanO2 cells (1 × 10^6^ cells/100 *μ*l per flank) were injected subcutaneously into the flanks to establish tumor-bearing mice. Two weeks after PanO2 cells were injected, drugs were administered by intraperitoneal injection for 2 weeks. The mice were randomly assigned to the following three groups: (1) control group, (2) anti-MCT1 and MCT4 (7ACC1, 0.3 mg/kg daily), and (3) anti-LDHA (FX-11, 2 mg/kg daily). Tumor size was measured every week for 28 days, and the formula (width^2^ × length)/2 was used to calculate tumor volume. Then, the mice were killed, and blood was collected for flow cytometric analysis. This study was approved by the Animal Ethical and Welfare Committee of NJMU (IACUC-1903002).

### 2.12. Statistical Analysis

Statistical analysis in the current study was performed using GraphPad Prism (version 6.0) and SPSS software (version 22.0). Quantitative data are presented as the mean (±SD). Differences in the mean between two groups were analyzed by Student's *t* test. The *χ*^2^ test was employed to analyze the associations of SIX1 or LDHA expression with clinicopathologic features. SIX1-LDHA interaction tests were performed using linear regression models. The Kaplan–Meier test was applied to calculate survival rates, and log-rank tests were used to examine differences in survival rates. *P* < 0.05 was considered to indicate statistical significance (^∗^*P* < 0.05, ^∗∗^*P* < 0.01, and ^∗∗∗^*P* < 0.001).

## 3. Results

### 3.1. SIX1 Promotes the Malignant Biological Behavior of PC

**Figure 1:**
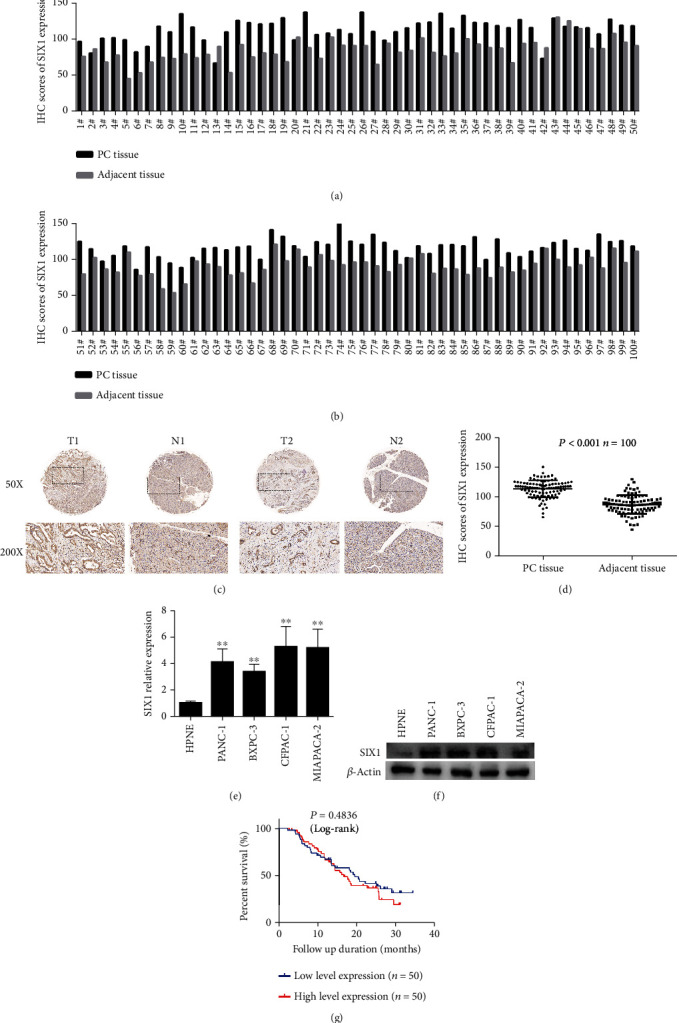
SIX1 expression in PC. (a, b) IHC scores for SIX1 expression in 100 pairs of PC tissues and adjacent tissues. (c) Representative IHC images of SIX1 expression in PC tissues and adjacent tissues. (d) According to IHC, SIX1 expression in PC tissues was significantly higher than that in the corresponding adjacent tissues. (e, f) Relative expression of SIX1 by qRT-PCR and western blotting in PC and HPNE cell lines; *β*-actin served as the internal control. (g) Kaplan–Meier curves showed no statistically significant effect of SIX1 expression on OS. The data are presented as the mean ± SD from three independent experiments. ^∗^*P* ≤ 0.05, ^∗∗^*P* ≤ 0.01, and ^∗∗∗^*P* ≤ 0.001.

SIX1 expression was detected in 100 pairs of PC and adjacent noncancerous pancreatic tissue samples by IHC. SIX1 expression was upregulated in 94 of the 100 PC samples (Figures 1(a) and 1(b)). Overall, SIX1 expression was significantly higher in PC tissues than in adjacent nonneoplastic tissues (Figures 1(c) and 1(d)). Furthermore, we examined SIX1 expression in a normal human pancreatic ductal cell line (HPNE) and PC cell lines (PANC-1, BXPC-3, CFPAC-1, and MIAPACA-2) by qRT-PCR and western blotting. As shown in Figures 1(e) and 1(f), the expression of SIX1 was higher in PANC-1, BXPC-3, CFPAC-1, and MIAPACA-2 cells than in HPNE cells.

Then, we analyzed the correlation between SIX1 expression and the clinicopathological characteristics of PC patients. The cutoff value for high/low SIX1 expression was determined by the median expression value based on IHC scores. As presented in Table 1, no significant association was observed between the SIX1 expression levels and gender, age, location, tumor differentiation, tumor-node-metastasis (TNM) stage, T stage, N stage, or major vascular invasion, but the SIX1 expression levels correlated positively with nerve infiltration and serum CA199 values (*P* = 0.046 and *P* = 0.029, respectively). However, the Kaplan–Meier survival curves showed that SIX1 had no influence on the overall survival (OS) of PC patients (Figure 1(g)). The above findings suggested that the expression of SIX1 is upregulated in PC cells and that SIX1 promotes the malignant biological behavior of PC.

### 3.2. SIX1 Promotes Lactate Accumulation in PC Cells and Leads to NK Cell Dysfunction

**Figure 2:**
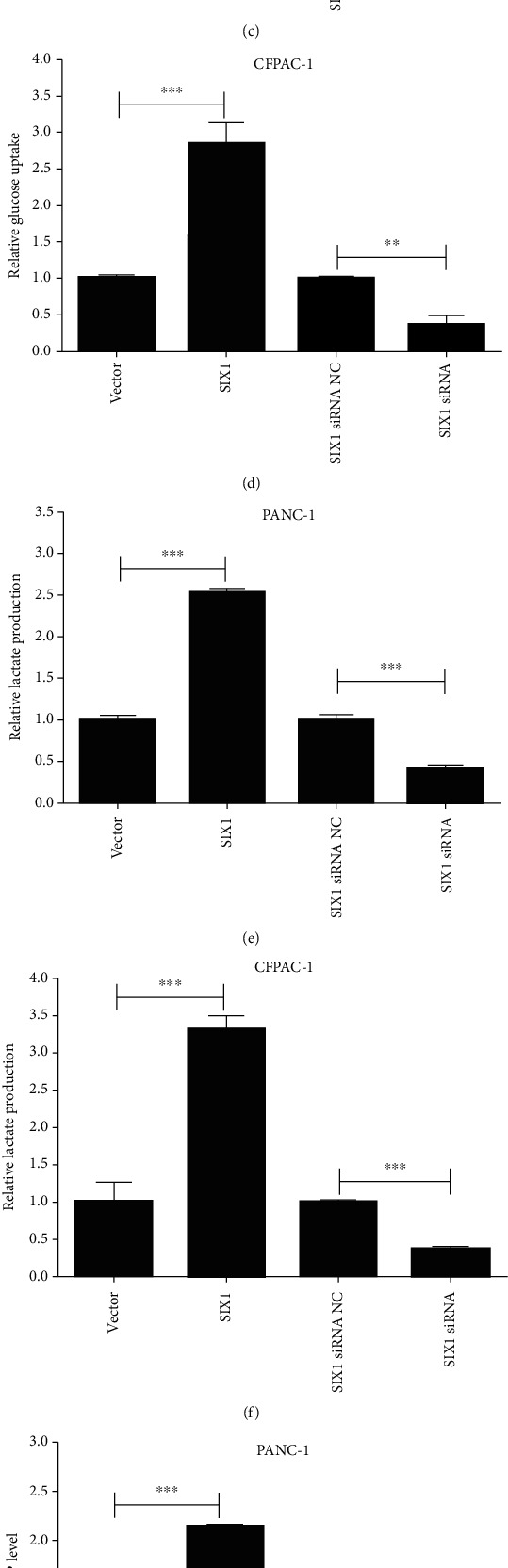
SIX1 promotes glycolysis in PC cells. (a, b) Relative expression of SIX1 by qRT-PCR and western blotting in PC cells with SIX1 overexpression vector and siRNA; *β*-actin served as the internal control. (c–h) Glucose consumption, lactate production, and ATP levels were measured in SIX1-overexpressing and SIX1 knockdown PC cells. The data are presented as the mean ± SD from three independent experiments. ^∗^*P* ≤ 0.05, ^∗∗^*P* ≤ 0.01, and ^∗∗∗^*P* ≤ 0.001.

To further investigate the function of SIX1, PANC-1 and CFPAC-1 cells were transfected with the SIX1 overexpression vector or siRNA. The transfection efficiency was assessed by qRT-PCR and western blotting (Figures 2(a) and 2(b)). As shown in Figures 2(c) and 2(d), SIX1 overexpression significantly increased glucose uptake in PANC-1 and CFPAC-1 cells, while knocking down SIX1 showed the opposite effect. Furthermore, lactate production and ATP levels in PC cells increased after SIX1 overexpression (Figures 2(e)–2(h)).

**Figure 3:**
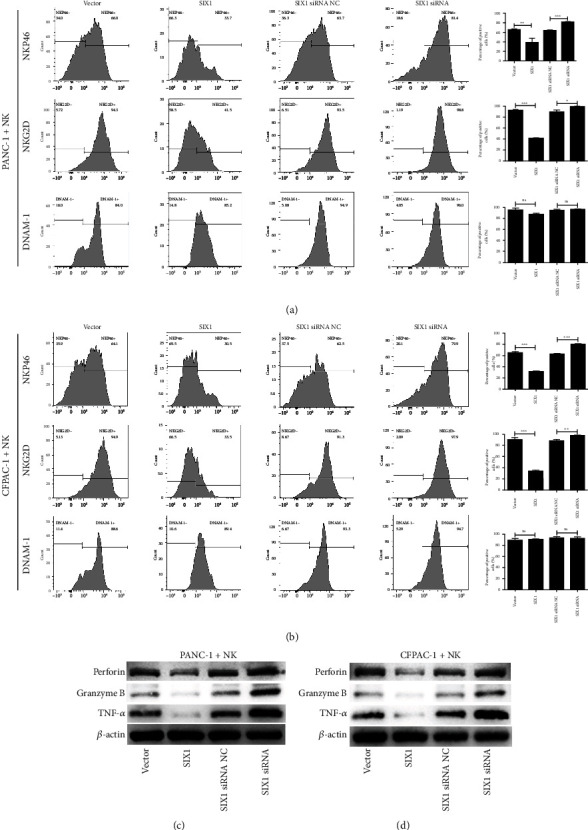
SIX1 overexpression in PC cells attenuates the function of NK cells. (a, b) The percentages of NKP46-, NKG2D-, and DNAM-1-positive NK cells cocultured with PANC-1 and CFPAC-1 cells. (c, d) Expression of perforin, granzyme B, and TNF-*α* in NK cells after coculture with PC cells according to western blotting; *β*-actin served as the internal control. The data are presented as the mean ± SD from three independent experiments. ^∗^*P* ≤ 0.05, ^∗∗^*P* ≤ 0.01, and ^∗∗∗^*P* ≤ 0.001.

To study the effect of SIX1 overexpression in PC cells on NK cell function, PC cells and NK cells were cocultured. As shown in Figures 3(a) and 3(b), the percentages of activating surface receptor (NKp46 and NKG2D)-positive NK cells were significantly lower after exposure to SIX1-overexpressing PANC-1 and CFPAC-1 cells, while SIX1 siRNA had the opposite effect. However, there were no significant changes in the percentages of DNAM-1-positive NK cells after the NK cells had been cultured with PANC-1 and CFPAC-1 cells. Furthermore, western blotting showed that NK cells expressed fewer cytotoxic mediators, including perforin, granzyme B, and TNF-*α*, after exposure to SIX1-overexpressing PANC-1 and CFPAC-1 cells, while SIX1 siRNA had the opposite effect (Figures 3(c) and 3(d)). The above results reveal that SIX1 promotes glycolysis in PC cells and that lactate accumulation results in NK cell dysfunction in PC.

### 3.3. LDHA Is a Functional Target of SIX1

**Figure 4:**
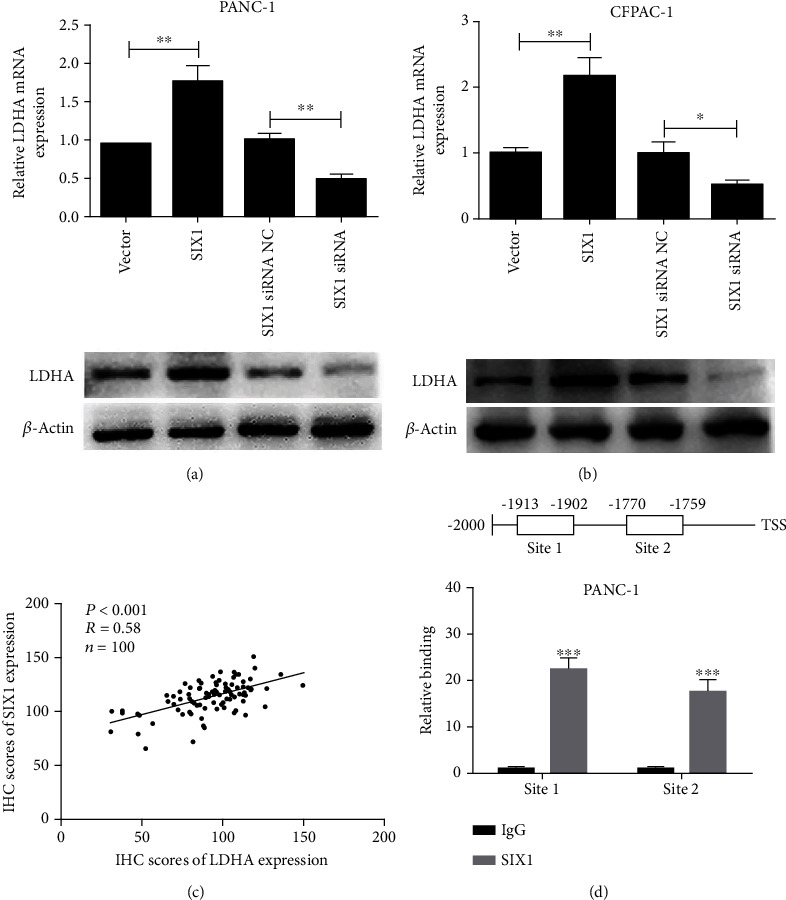
SIX1 directly targets LDHA in PC. (a, b) LDHA expression levels in SIX1-overexpressing or SIX1 knockdown cells were measured by qRT-PCR and western blotting, and *β*-actin served as the internal control. (c) In human PC tissues, LDHA was positively correlated with SIX1 expression. (d) A ChIP-qPCR assay demonstrated the direct binding of SIX1 to the LDHA promoter in PANC-1 cells. The data are presented as the mean ± SD from three independent experiments. ^∗^*P* ≤ 0.05, ^∗∗^*P* ≤ 0.01, and ^∗∗∗^*P* ≤ 0.001.

To identify downstream effectors of SIX1, the relationship between SIX1 and 16 glycolysis-related genes was analyzed using The Cancer Genome Atlas (TCGA) database. As shown in Figure S1, HK1, PKM2, and LDHA showed a strong correlation with SIX1 (*P* < 0.001, *r* > 0.4). We further studied the relationship between SIX1 and LDHA expressions, as measured by qRT-PCR and western blotting, SIX1 overexpression led to increased LDHA expression, and SIX1 knockdown led to decreased LDHA expression in PANC-1 and CFPAC-1 cells (Figures 4(a) and 4(b)). We then analyzed the expression of LDHA and SIX1 in PC tissues by IHC. As shown in Figure 4(c), LDHA protein expression correlated positively with SIX1 expression (*P* < 0.001, *r* = 0.58). In addition, ChIP-qPCR revealed that the LDHA promoter region in the two predicted binding sites exhibited significant enrichment after immunoprecipitation with an anti-SIX1 antibody (Figure 4(d)).

### 3.4. Upregulation of LDHA Is Associated with Poor Outcomes in PC

**Figure 5:**
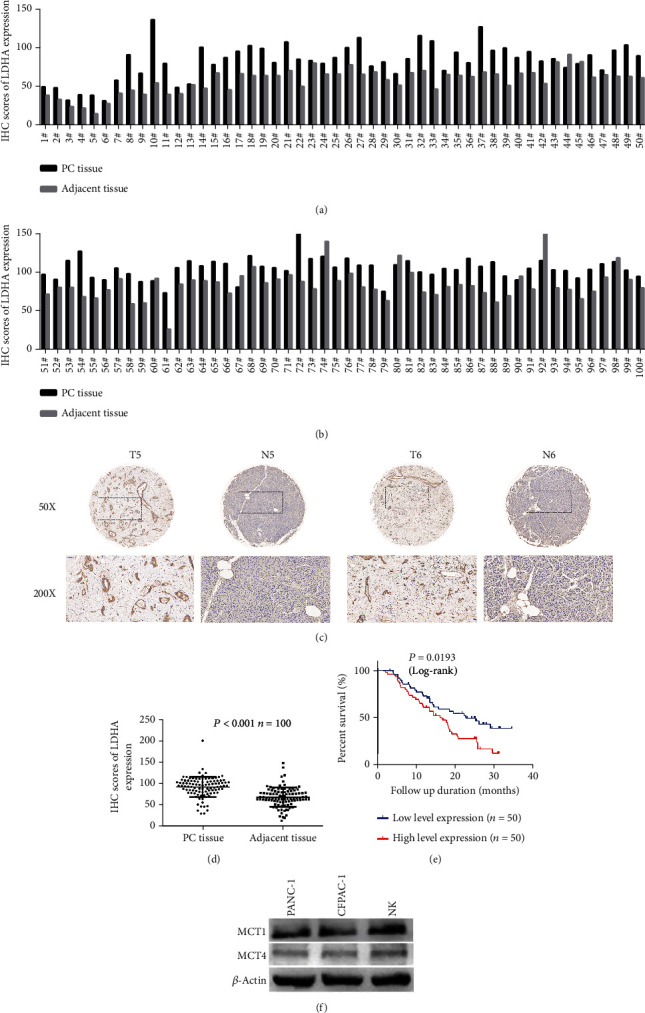
LDHA expression in PC. (a, b) IHC scores of LDHA expression in 100 pairs of PC tissues and adjacent tissues. (c) Representative IHC images of LDHA expression in PC tissues and adjacent tissues. (d) According to IHC, LDHA expression in PC tissues was significantly higher than that in corresponding adjacent tissues. (e) Kaplan–Meier curves comparing the OS of patients grouped by LDHA expression. (f) MCT1 and MCT4 expression levels in PANC-1, CFPAC-1, and NK cells were measured by western blotting, and *β*-actin served as the internal control. The data are presented as the mean ± SD from three independent experiments. ^∗^*P* ≤ 0.05, ^∗∗^*P* ≤ 0.01, and ^∗∗∗^*P* ≤ 0.001.

IHC was performed to detect LDHA expression in 100 pairs of PC and adjacent nontumor pancreatic tissue samples. LDHA expression was upregulated in 91 of the 100 PC samples (Figures 5(a) and 5(b)). Overall, LDHA expression was significantly higher in PC tissues than in adjacent nonneoplastic tissues (Figures 5(c) and 5(d)).

Then, we analyzed the correlation between LDHA expression and the clinicopathological characteristics of PC patients. The cutoff value for high/low LDHA expression was the median expression value based on IHC scores. As presented in Table 1, no significant association was observed between LDHA expression levels and gender, age, location, tumor differentiation, N stage, nerve invasion, or major vascular invasion, but LDHA expression levels correlated positively with TNM stage, T stage, and serum CA199 values (*P* = 0.045, *P* = 0.001, and *P* = 0.029, respectively). The Kaplan–Meier survival curves showed that patients with higher LDHA expression had lower overall postoperative survival (Figure 5(e)). In summary, our data show that LDHA is highly expressed in PC and is closely related to clinicopathological features and prognosis.

### 3.5. LDHA-Mediated Lactate Production in PC Cells Promotes NK-Cell Dysfunction

**Figure 6:**
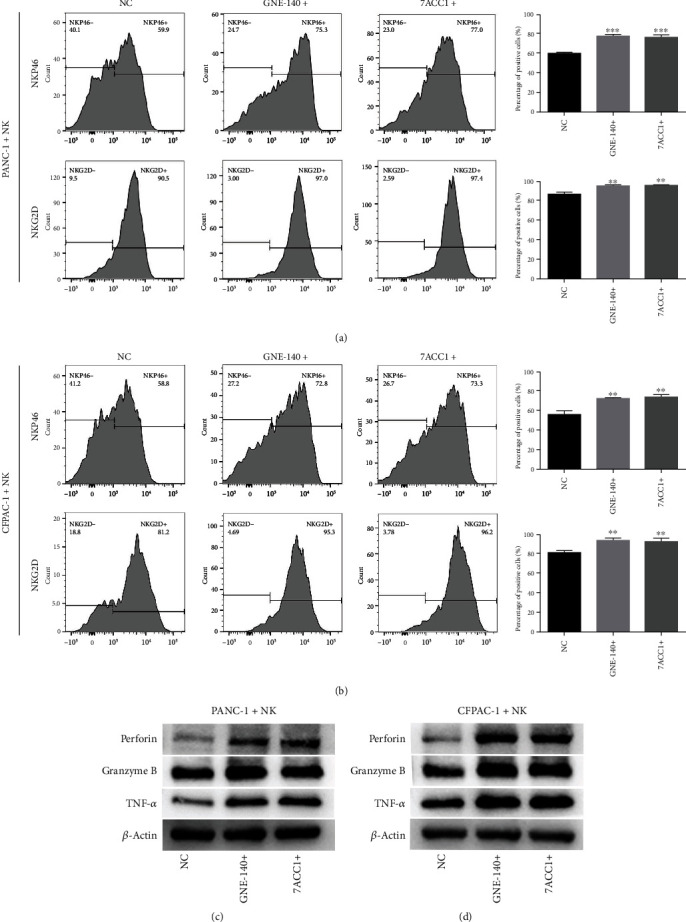
LDHA expression in PC cells affects the function of NK cells. (a, b) The percentages of NKP46- and NKG2D-positive NK cells cocultured with PC cells treated with GNE-140 or 7ACC1. (c, d) Expression of perforin, granzyme B, and TNF-*α* in NK cells cocultured with PC cells treated with GNE-140 or 7ACC1 according to western blotting; *β*-actin served as the internal control. The data are presented as the mean ± SD from three independent experiments. ^∗^*P* ≤ 0.05, ^∗∗^*P* ≤ 0.01, and ^∗∗∗^*P* ≤ 0.001.

The main function of LDHA is to catalyze the formation of lactate from pyruvate. Therefore, we detected the expression of lactate transporters in PC cells and NK cells through western blotting (Figure 5(f)). Next, we used an LDHA inhibitor (GNE-140) and a lactate transporter (MCT)-specific blocker (7ACC1) to inhibit the production and transport of lactate. As shown in Figures 6(a) and 6(b), the percentages of NKG2D- and NKp46-positive NK cells were significantly higher after PANC-1 and CFPAC-1 cells were exposed to GNE-140 and 7ACC1 (Figures 6(a) and 6(b)). Furthermore, western blotting showed that NK cells expressed more perforin, granzyme B, and TNF-*α* after cocultured with GNE-140- and 7ACC1-treated PC cells (Figures 6(c) and 6(d)). The above results revealed that LDHA-mediated lactate production in PC cells promotes NK cell dysfunction.

### 3.6. LDHA Can Partially Restore the Effect of SIX1 in PC

**Figure 7:**
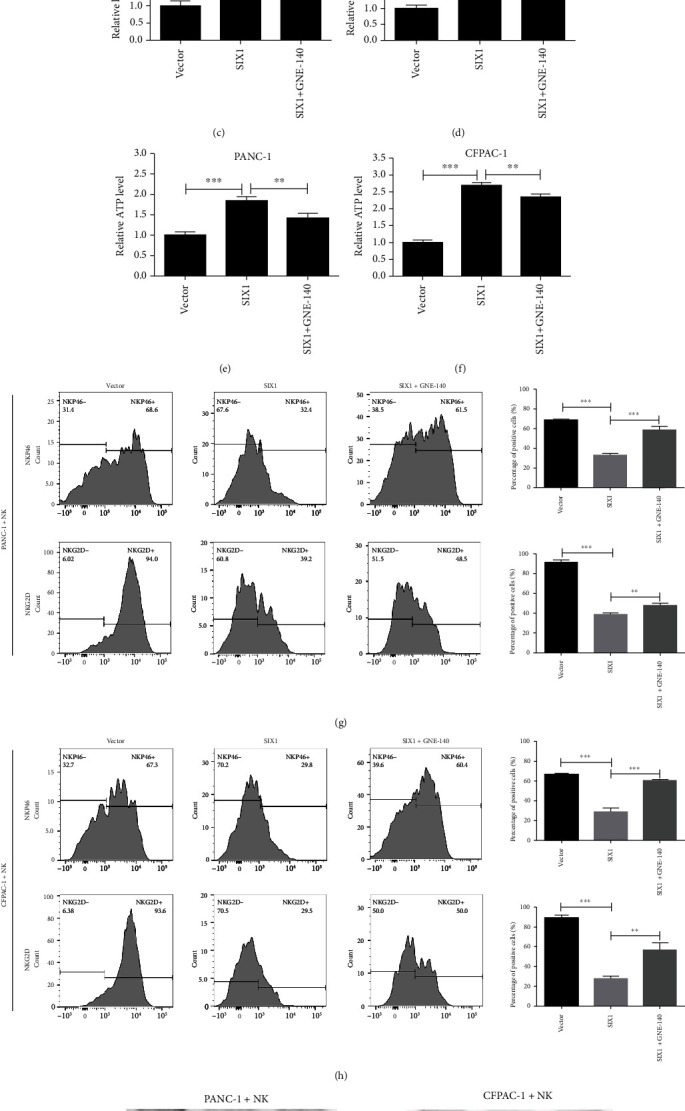
LDHA can partially restore the effects of SIX1 in PC. (a–f) Glucose consumption, lactate production, and ATP levels were measured to analyze glycolysis in SIX1-overexpressing cells treated with an LDHA inhibitor. (g, h) The percentages of NKP46- and NKG2D-positive NK cells cocultured with SIX1-overexpressing PC cells treated with GNE-140. (i, j) Expression of perforin, granzyme B, and TNF-*α* in NK cells cocultured with SIX1-overexpressing PC cells treated with GNE-140 as indicated by western blotting; *β*-actin served as the internal control. The data are presented as the mean ± SD from three independent experiments. ^∗^*P* ≤ 0.05, ^∗∗^*P* ≤ 0.01, and ^∗∗∗^*P* ≤ 0.001.

To further investigate whether LDHA is involved in the role of SIX1 in PC, LDHA inhibitor was used to treat SIX1-overexpressing PANC-1 and CFPAC-1 cells. The results showed that the increase in glycolysis capacity induced by SIX1 overexpression was recovered by the LDHA inhibitor (Figures 7(a)–7(f)). The decreased percentages of NKG2D- and NKp46-positive NK cells cocultured with SIX1-overexpressing PC cells were recovered with GNE-140 treatment (Figures 7(g) and 7(h)). Furthermore, western blotting showed that NK cells cocultured with SIX1-overexpressing PC cells treated with GNE-140 expressed lower levels of cytotoxicity mediators than the SIX1-overexpressing group (Figures 7(i) and 7(j)). These results indicated that LDHA, as a functional target of SIX1, may play an important role in promoting glycolysis and NK cell dysfunction under conditions of SIX1 overexpression.

### 3.7. LDHA-Mediated Lactate Production Promotes NK Cell Dysfunction In Vivo

**Figure 8:**
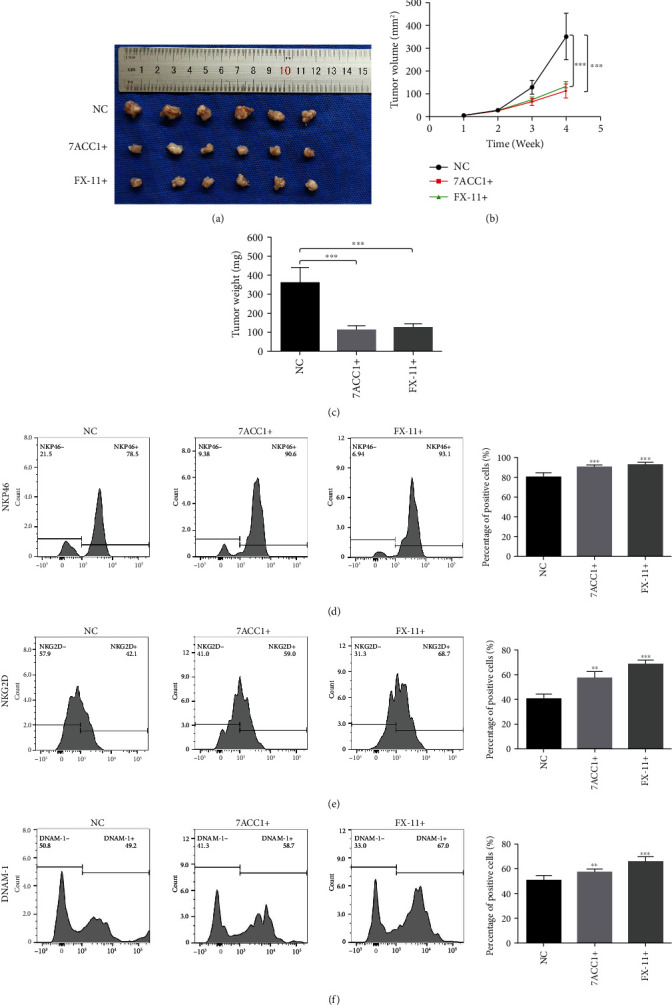
Lactate production mediated by LDHA attenuates the function of NK cells in vivo. (a) Tumors were obtained from C57BL/6 mice treated with 7ACC1 or FX-11 by intraperitoneal injection. (b) The growth curve of tumor volume from C57BL/6 mice. (c) The average weight of tumors from C57BL/6 mice treated with 7ACC1 or FX-11 by intraperitoneal injection. (d–f) The percentages of NKG2D-, NKp46-, and DNAM-1-positive NK cells in the blood of C57BL/6 mice treated with 7ACC1 or FX-11 by intraperitoneal injection. The data are presented as the mean ± SD from three independent experiments. ^∗^*P* ≤ 0.05, ^∗∗^*P* ≤ 0.01, and ^∗∗∗^*P* ≤ 0.001.

PanO2 cells were implanted into C57BL/6 mice subcutaneously, and we measured the sizes of the xenograft tumors every 7 days for 28 days. As shown in Figures 8(a)–8(c), the tumor volumes were significantly smaller, and the tumor weights were significantly reduced after treatment with a lactate transporter blocker (7ACC1) and LDHA inhibitor (FX-11) compared with those in the control group. Furthermore, the percentages of NKG2D-, NKp46-, and DNAM-1-positive NK cells in the blood were significantly higher after intraperitoneal injection of 7ACC1 or FX-11 (Figures 8(d)–8(f)). Taken together, these results demonstrate that LDHA-mediated lactate production promotes NK cell dysfunction in PC.

## 4. Discussion

Glycolysis is a key feature of cancer, and lactate, as a product of glycolysis, plays an important role in the malignant progression of tumors [14, 28]. The innovative discovery of this study is that the SIX1/LDHA axis may mediate the upstream regulatory mechanism of lactate and that lactate produced by glycolysis in PC has an inhibitory effect on NK cell function.

As a transcription factor, SIX1 participates in the development of normal tissues by regulating cell proliferation, apoptosis, or differentiation [29]. Studies have shown that the increased expression of SIX1 in tumors, such as in hepatocellular carcinoma [30], breast cancer [31], and colorectal cancer [32], often indicates poor clinical outcomes and promotes tumor proliferation, invasion, and metastasis. Recent studies have shown that SIX1 also promotes cancer progression by promoting glycolysis. Li et al.'s research shows that SIX1 directly increases the expression of many glycolytic genes, promoting the Warburg effect and tumor growth in vitro and in vivo [11]. However, whether and how SIX1 affects PC development and progression remain unclear. This study found that SIX1 expression was increased in PC tissues and cell lines. Clinical data analysis showed that PC patients with high SIX1 expression had higher levels of nerve invasion and serum CA19-9 expression than those with low SIX1 expression. Moreover, SIX1 overexpression increased glucose uptake and lactate and ATP production in PC cells. The small sample size may be the reason Kaplan–Meier survival curve analysis showed that SIX1 had no influence on the survival of PC patients.

To identify SIX1 downstream effectors, we analyzed the correlations between SIX1 and some main glycolysis-related genes through TCGA database. We found that SIX1 is positively correlated with many main glycolysis genes and that SIX1 may be a key regulator of glycolytic gene expression. LDHA attracted our attention because it is not only a commonly used clinical test index but also shows a strong correlation with NK cell function [18, 33]. In this study, we analyzed the relationship between SIX1 and LDHA. According to the immunohistochemical results of the tissue microarray, we found that LDHA expression is positively correlated with SIX1 expression. Moreover, through ChIP experiments, we found that SIX1 can directly bind to the promoter region of LDHA. Further study revealed that LDHA expression in cancer tissues of patients with PC is higher than that in adjacent tissues, and patients with high LDHA expression have a worse prognosis than those with low expression. Functional recovery experiments showed that SIX1 has a direct positive regulatory effect on LDHA. LDHA is an important enzyme in the glycolysis pathway that catalyzes the formation of lactate from pyruvate in the acidic microenvironment of cancer. Studies have shown that glycolysis has an important impact on tumor immunity. Cancer cells are more prone to glycolytic metabolism, which leads to the depletion of extracellular glucose and the release of large amounts of lactic acid, but this leads to tumor-infiltrating T cell dysfunction [34]. Similarly, pancreatic tumors that overexpress the lactate exporter MCT4 have a negative impact on the surrounding immune environment milieu [35]. These studies usually emphasize that glycolysis or lactic acid inhibits the immune function of T cells, but there are relatively few studies on NK cells.

NK cells are mainly distributed in peripheral blood, accounting for approximately 5-15% of PBMCs [36, 37]. Activated NK cells can synthesize and secrete a variety of killing mediators, such as perforin, granzyme B, and TNF-*α*, which can regulate immunity and directly kill tumor cells [38]. This study revealed a new mechanism by which glycolytic metabolism in PC affects NK cell function. In this study, we cocultured PC cells and NK cells. After SIX1 overexpression in PC cells, the percentages of activating surface receptor (NKG2D and NKp46)-positive NK cells decreased, and the secretion of perforin, granzyme B, and TNF-*α* decreased. 7ACC1 is a blocker of MCT1 and MCT4 and thus is a blocker of lactate transport [39, 40]. In the experiment, we used 7ACC1 to inhibit MCT1 and MCT4 in a coculture system. The results showed that the function of NK cells was enhanced after 7ACC1 treatment, and the inhibitory effect of SIX1 on NK cells was partially reversed by 7ACC1. Similarly, the inhibitory effect of SIX1 on NK cells was partially restored by treatment with the LDHA inhibitor GEN140. The above results indicate that the inhibitory effect of PC on NK cells is mediated by lactate produced via the SIX1/LDHA axis. Furthermore, we also generated a PC model in mice. Flow cytometry was used to detect peripheral blood NK cells in mice treated with 7ACC1 and the LDHA inhibitor FX-11, and we found that the function of NK cells in peripheral blood was enhanced. In summary, we can conclude that lactate produced via the SIX1/LDHA axis is likely to localize to NK cells and act as a signaling molecule to mediate NK cell dysfunction. However, the specific mode of action of lactate in NK cells is still unclear and needs further study. Recently, researchers have discovered lactate-derived histone lysine lactylation as a new epigenetic modification [41, 42]. Whether lactate affects the function of NK cells through this pathway is also worthy of in-depth study.

## 5. Conclusion

In summary, this study comprehensively discussed the important role of the SIX1/LDHA axis in cell metabolism and NK cell dysfunction in PC and indicated that it has the potential to become a new target for the treatment of PC.

## Figures and Tables

**Table 1:** Correlation between clinicopathological features and expression of SIX1 and LHDA.

Characteristics	SIX1 expression		*P* value	LDHA expression		*P* value
	Low expression (*n* = 50)	High expression (*n* = 50)		Low expression (*n* = 50)	High expression (*n* = 50)	
Gender			0.663			0.19
Male	34	36		32	38	
Female	16	14		18	12	
Age (years)			0.081			1
<60	19	11		15	15	
≥60	31	39		35	35	
Location			0.832			0.137
Head	34	33		37	30	
Body+tail	16	17		13	20	
Tumor differentiation			0.673			1
G1+G2	34	32		33	33	
G3	16	18		17	17	
TNM stage			0.422			0.045^∗^
I+II	29	25		32	22	
III+IV	21	25		18	28	
T stage			0.316			0.001^∗∗^
T1+T2	29	24		35	18	
T3+T4	21	26		15	32	
N stage			0.418			0.685
N0	23	19		22	20	
N1+N2	27	31		28	30	
Nerve invasion			0.046^∗^			0.505
No	8	2		4	6	
Yes	42	48		46	44	
Serum CA19-9 (U/ml)			0.029^∗^			0.029^∗^
<39	12	4		12	4	
≥39	38	46		38	46	
Major vascular invasion			1			0.075
No	14	14		18	10	
Yes	36	36		32	40	

Note: ^∗^*P* < 0.05, ^∗∗^*P* < 0.01, and ^∗∗∗^*P* < 0.001. G1 stands for well differentiation, G2 stands for moderately differentiation, and G3 stands for poor differentiation.

## Data Availability

Please contact the author for data request.

## Abbreviations

PC: Pancreatic cancer
SIX1: Sine oculis homeobox homolog 1
ChIP: Chromatin immunoprecipitation
LDHA: Lactate dehydrogenase A
PBMC: Peripheral blood mononuclear cell
IHC: Immunohistochemistry
MDSCs: Myeloid-derived suppressor cells.

## Supplementary Materials

Supplementary 1 Figure S1: (A–P) the relationships between SIX1 and GK, HK1, GPI, PFKL, ALDOA, ALDOB, TPI1, GAPDH, PGK1, PGAM1, ENO1, ENO2, PKLR, PKM2, LDHA, or LDHB according to the TCGA database.

Supplementary 2 Table S1: list of the oligonucleotide primers used for amplification.
