# Supplementary figures and images for: The SIX1/LDHA Axis Promotes Lactate Accumulation and Leads to NK Cell Dysfunction in Pancreatic Cancer
Source: J Immunol Res. 2023 Mar 8;2023:6891636. doi: 10.1155/2023/6891636 (PMC10022590; doi:10.1155/2023/6891636)

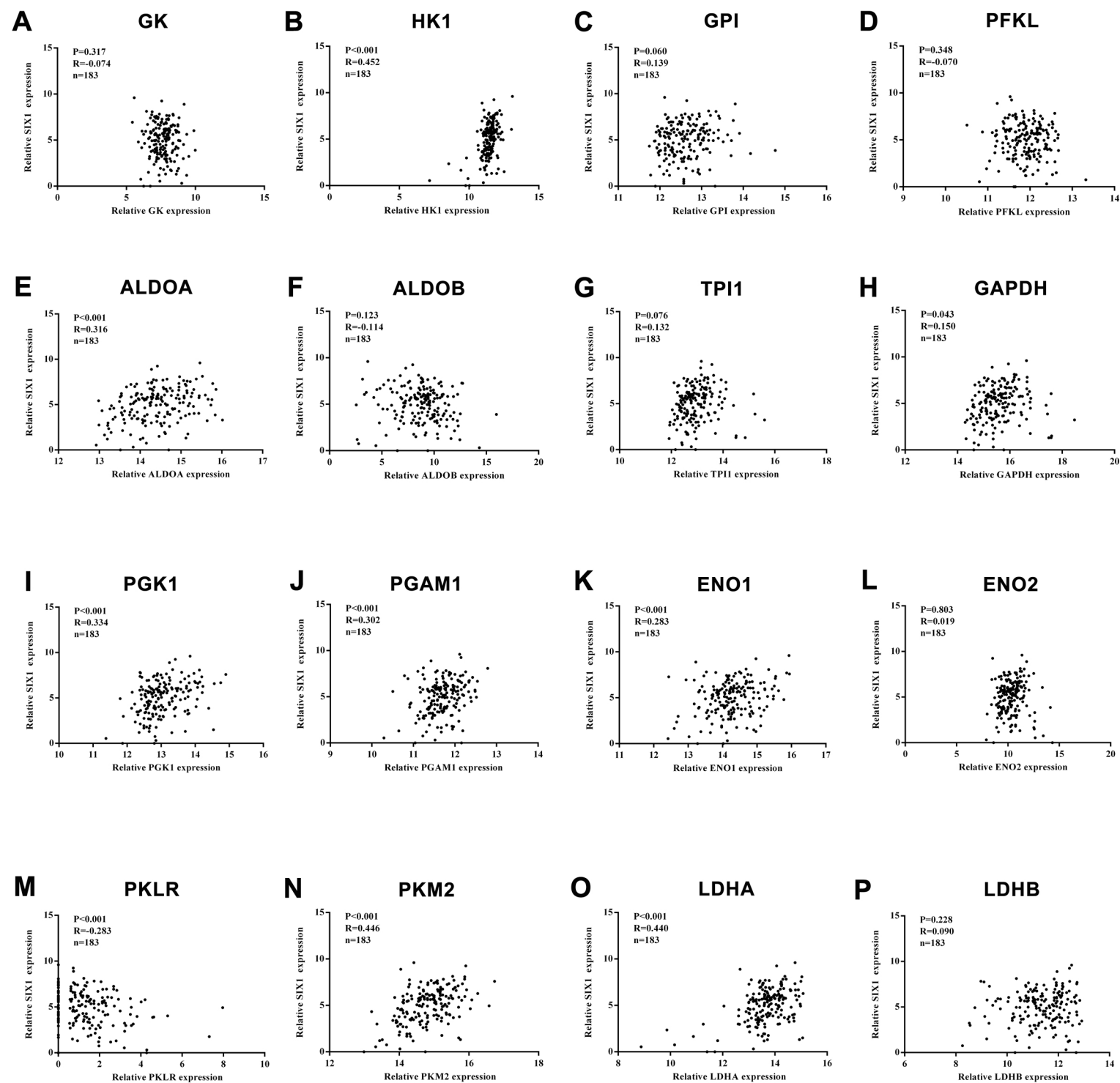

Supplement: Supplementary 1 — Figure S1: (A–P) the relationships between SIX1 and GK, HK1, GPI, PFKL, ALDOA, ALDOB, TPI1, GAPDH, PGK1, PGAM1, ENO1, ENO2, PKLR, PKM2, LDHA, or LDHB according to the TCGA database. [file 6891636.f1.pdf]
