# Supplementary material for: The SIX1/LDHA Axis Promotes Lactate Accumulation and Leads to NK Cell Dysfunction in Pancreatic Cancer
Source: J Immunol Res. 2023 Mar 8;2023:6891636. doi: 10.1155/2023/6891636 (PMC10022590; doi:10.1155/2023/6891636)
Supplement: Supplementary 2 — Table S1: list of the oligonucleotide primers used for amplification. [file 6891636.f2.docx]

Supplementary Table 1. List of the oligonucleotides primers used for amplification

| Experiment | Genes | Sequence(5’-3’) |
| --- | --- | --- |
| qRT-PCR | SIX1 | F: 5’-CGCGCACAATCCCTACCCATCGCC -3’ |
|  |  | R: 5’-CTTCCAGAGGAGAGAGTTGGTTCTG-3’ |
|  | LDHA | F: 5’-ATGGCAACTCTAAAGGATCAGC-3’ |
|  |  | R: 5’-CCAACCCCAACAACTGTAATCT -3’ |
|  | β-actin | F: 5’- ATTGCCGACAGGATGCAGAA-3’ |
|  |  | R: 5’- GCTGATCCACATCTGCTGGAA-3’ |
| ChIP qPCR | Site1 | F: 5’- CTTCTGCACACCTCTTCCCA -3’ |
|  |  | R: 5’- TCTCACCTCAAACACACAGCT -3 |
|  | Site2 | F: 5’- GCCCTGAGGTACTCTGAAGAA -3’ |
|  |  | R: 5’- TGACTCATGCCTGTAATCCCA -3’ |
